# Stationary DNA Origami Register Drives Fast Sequential DNA Computing

**DOI:** 10.1021/acscentsci.4c02006

**Published:** 2024-12-11

**Authors:** Pu Deng, Jiahao Lin, Wei Sun

**Affiliations:** Key Laboratory for the Physics and Chemistry of Nanodevices, School of Electronics, Peking University, Beijing, China, 100871

Unlike semiconductor electronics, DNA computing leverages the unique base-pairing and recognition specificity of DNA strands to perform complex information processing at the molecular level.^1−4^ Early demonstrations highlighted DNA’s potential for parallel computation and information storage in chaotic liquid environments, motivating exploration beyond silicon chips.^2^ Research soon transitioned to functional systems, such as logical gates and molecular machines.^3,4^ Currently, DNA computing has applications in fields like information processing, molecular robotics, therapeutics, etc.,^5−9^ driven by biocompatibility and computation efficiency. However, increasing computational complexity and scalability of DNA computing remain experimentally challenging, owing to its unconfined diffusion-based mechanism. The use of DNA origami registers in a computing reaction solution offers spatial confinement and addressability to store information and direct DNA circuit interactions, resulting in reduced signal decay and interference. This marks an exciting step forward in the case of computation complexity.^10^ Yet, reliance on solution-based data transfer remains an obstacle for the practical application of DNA computing, especially when multiple steps are involved.

**Figure 1:**
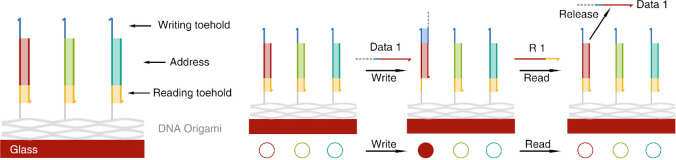
Mechanism of signal writing and reading with solid-state DNA origami registers. Reproduced with permission from ref (1). Copyright 2024 American Chemical Society.

In this issue of *ACS Central Science*, Fan, Wang, Lv, Jia, and co-workers present a novel approach to address these issues by creating a solid-state DNA origami register.^1^ Specifically, the authors integrate solid-state DNA origami registers, which are affixed to a glass surface, to liquid-phase DNA circuits using strand displacement reactions (Figure 1). The registers remain stationary while data are written, read, and rewritten. Through this innovative architecture, not only does the register retain the complexity of liquid-phase molecular computation, but it also integrates rapid molecular interactions at the liquid–solid interface. As a result, this design simplifies the need for physical movement in liquid-phase DNA origami registers and reduces the data transmission time from several hours to less than an hour.

**Figure 2:**
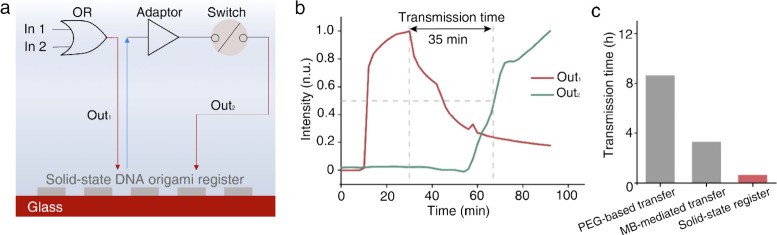
Solid-state DNA origami registers reduce the transmission time to less than an hour. a. Schematic illustration of a two-layer circuit connected to the solid-state DNA origami register. b. Dynamics of Out1 and Out2 signal intensity changes during circuit operation. The solid-state DNA origami register improves the transmission time to 35 min. c. Comparison of transmission time for solid-state DNA origami register versus PEG- or MB-mediated transfer of registers. Reproduced with permission from ref (1). Copyright 2024 American Chemical Society.

Furthermore, by introducing mismatches at three distinct positions (L, M, and R), the authors engineer the free energy and conformation of DNA molecules used in the Converter for accelerating downstream signal processing. The amplification rates of all three Converters have been largely improved compared to the original design, and the M Convertor presents the best signal-to-noise ratio. The adaptor with the M Convertor converts and amplifies 1 nM trace signals by 100-fold, significantly breaking the previous amplification limit of 10 nM. Finally, this work successfully demonstrates a two-layer sequential circuit, with an OR gate feeding into a switch, showcasing the ability of this integrated system to execute complex DNA computation rapidly and reliably (Figure 2a).

This work tackles the diffusion-limited computation challenge in DNA computing by leveraging a solid-state DNA origami register to improve speed and sensitivity. By reducing data transmission time and enhancing signal amplification, the system achieves a significant boost in overall computational efficiency (Figure 2b and c). Building on this foundation, a two-layer logic circuit was constructed, showcasing the scalability and versatility of the system for advanced DNA computations.

Despite the improvements, some limitations remain. This system is highly dependent on stable environmental conditions, such as temperature and buffer composition, to ensure correct DNA strand interactions. Deviations in these conditions may lead to data loss or misinterpretation. Moreover, while the two-layer sequential circuit demonstrated here serves as an effective proof of concept, further research is necessary to assess its scalability for more complex circuits and computational tasks.

Ultimately, this study marks a significant milestone in the evolution of DNA-based computational systems, highlighting the remarkable potential for future advancements. Specifically, by introducing stationary, rewritable DNA registers on a solid-state platform, it lays the groundwork for the development of more compact and scalable DNA computing systems. Additionally, simplifying data flow through immobilized registers while eliminating the need for moving storage elements offers a clear path for integration with microfluidic systems, paving the way for increased automation. Furthermore, the use of DNA origami with precise nanometer-scale features enables the precise positioning of heterogeneous molecules. Coupled with real-time, single-molecule fluorescence imaging, this approach allows detailed observation of addressable storage and release dynamics, providing a solid foundation for high-throughput analysis of molecular interactions and functional dynamics. These advances are particularly inspiring toward the creation of next-generation miniaturized DNA devices, with potential applications in nanotechnology, biomedical engineering, and other domains requiring molecular-level calculations and data storage.
